# Direct Magnetoelectric Effect in a Sandwich Structure of PZT and Magnetostrictive Amorphous Microwires

**DOI:** 10.3390/ma13040916

**Published:** 2020-02-19

**Authors:** Abdulkarim Amirov, Irina Baraban, Larissa Panina, Valeria Rodionova

**Affiliations:** 1 Laboratory of Novel Magnetic Materials & Institute of Physics Mathematics and Informational Technologies, Immanuel Kant Baltic Federal University, 236016 Kaliningrad, Russia; irinmachay@gmail.com (I.B.); drlpanina@gmail.com (L.P.); valeriarodionova@gmail.com (V.R.); 2 Amirkhanov Institute of Physics Daghestan Scientific Center, Russian Academy of Sciences, 367003 Makhachkala, Russia; 3 Institute of Novel Materials and Nanotechnology, National University of Science and Technology MISiS, 119049 Moscow, Russia

**Keywords:** multiferroics, magnetoelectric composite, magnetoelectric effect, amorphous ferromagnetic microwires

## Abstract

The magnetoelectric (ME) response in a trilayer structure consisting of magnetostrictive Fe_77.5_B_15_Si_17.5_ amorphous microwires between two piezoelectric PZT (PbZr_0.53_Ti_0.47_O_3_) layers was investigated. Soft magnetic properties of wires make it possible to operate under weak bias magnetic fields below 400 A/m. Enhanced ME voltage coefficients were found when the microwires were excited by ac magnetic field of a frequency of 50–60 kHz, which corresponded to the frequency of electromechanical resonance. The as-prepared microwires were in a glass coat creating a large thermoelastic stress and forming a uniaxial magnetic anisotropy. The effect of glass-coat removal and wire annealing on ME coupling was investigated. The glass coat not only affects the wire magnetic structure but also prevents the interfacial bonding between the electric and magnetic subsystems. However, after its removal, the ME coefficient increased slightly less than 10%. Refining the micromagnetic structure and increasing the magnetostriction by stress release during wire annealing (before or after glass removal) strongly increases the ME response up to 100 mV/(cm × Oe) and reduces the characteristic DC magnetic field down to 240 A/m. Although the achieved ME coefficient is smaller than reported values for multilayered films with layers of PZT and soft magnetic alloys as Metglass, the proposed system is promising considering a small volume proportion of microwires.

## 1. Introduction

Composite multiferroics have recently drawn considerable interest as promising multifunctional materials owing to large magnetoelectric (ME) coupling [1,2]. In the direct ME effect, the electric polarization or voltage can be changed or generated by an applied magnetic field:

(1) $$E=\alpha_{ME}\Delta H$$

(2) $$\alpha_{ME}=\frac{\Delta V}{b\;\Delta H}$$

In Equations (1) and (2), $\alpha_{ME}$ is the magnetoelectric coefficient, ΔV is the voltage output produced by changing the applied magnetic field ΔH and b is the sample thickness. Natural single-phase multiferroics based on complex micromagnetic structure show giant ME effect but typically at low temperatures [3]. In contrast, multiferroic composites, which consist of piezoelectric and magnetostrictive phases, demonstrate giant ME response at room temperatures as a result of cross-interactions between the both phases [4]. Then, the ME coefficient strongly depends on the condition of ferromagnetic–ferroelectric interfaces. For this reason, layered ferromagnetic/ferroelectric structures are efficient ME materials at room temperatures [5,6]. Among them, traditional Terfenol-D laminates with different piezoelectric materials demonstrate giant ME effect but they are not suitable for many applications where the use of high magnetic fields is prohibited due to its low magnetic susceptibility and high saturation fields. Current research on magnetoelectric composites has focused on search of new magnetostrictive materials having balanced soft magnetic and magnetostrictive properties. Amorphous alloy metglass typically produced in the form of ribbons is attractive for achieving large ME response at weak magnetic fields.

Among soft magnetic materials, amorphous glass-coated microwires are actively studied as promising functional materials for applications in sensors and smart composites [7]. It is important to emphasize that their micromagnetic structure and magnetization reversal strongly correlate with the magneto mechanical interactions determined by the magnetostriction and internal stress [8]. At the same time, the magnitude of the magnetostriction of Fe-based microwires is relatively high (up to $10^{-5}$). Therefore, they can be used as components for multiferroic structures as proposed in the present work.

The structure of two-phase ME composites can be described considering the concept of phase connectivity [1,2]. The connectivity schemes are typically denoted as 0-3, 2-2, and 1-3 where the numbers specify the connectivity of phases. Magnetostrictive wires can be implemented as 2-2 type laminates (layers of magnetostrictive wires between the layers of ferroelectric material) and 1-3 type composites (magnetostrictive wires embedded into the ferroelectric matrix). The structure considered here is of the 2-2 type with the internal layer of closely spaced microwires between two piezoelectric layers. We have demonstrated that high ME coefficients are obtained at weak bias magnetic fields, which correlates with the magnetization reversal of the microwires. For this reason, the proposed tri-layered ME sandwich has potential applications in low magnetic field sensors, in particular, for biomedical applications [9].

## 2. Materials and Methods 

We used the 2-2 type of the connectivity scheme typical of layered composites. This type may be further subdivided to other related types with modified connectivity between piezoelectric and magnetic layers. The choice of connectivity of subtypes depends on the needed mode of ME interaction.

**Figure 1:**
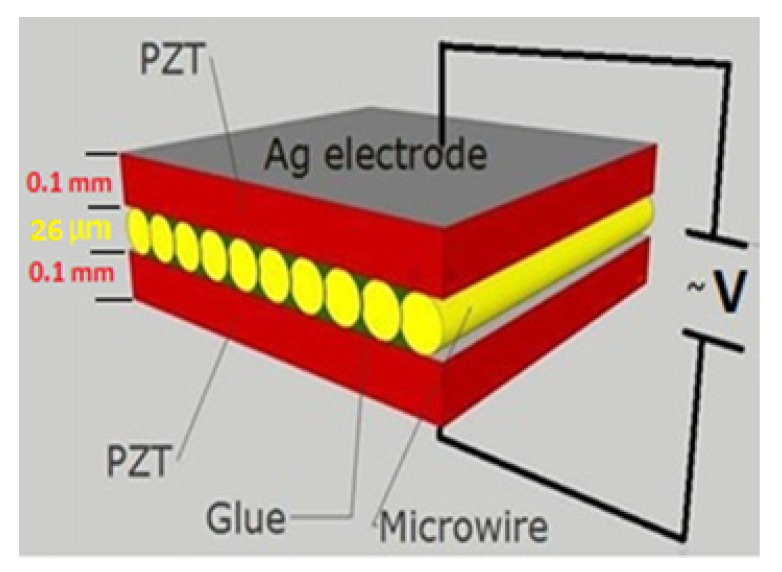
Sketch of the structure of magnetoelectric (ME) composite with microwires.

Simple three-layer multiferroic P-M-P type composites with piezoelectric (P) and magnetostrictive (M) components were fabricated. The magnetoelectric sandwich consisted of Fe_77.5_B_15_Si_7.5_ amorphous glass-coated microwires as a magnetic component and two layers of PbZr_0.53_Ti_0.47_O_3_ (PZT) with thicknesses of 0.1 mm each as a piezoelectric component. The ME sandwich was fabricated by bonding with the use of special adhesive (GE varnish). For this procedure, the disk of commercial PZT ceramic (JSC Research Institute “ELPA”, Zelenograd, Russia) was cut into two rectangular plates of the required dimensions and cleaned in ultrasonic bath. Then, bonded sides of PZT plates were covered by adhesive and the magnetostrictive microwires were mounted onto one PZT plate in parallel to each other. The alignment of microwires on PZT layer was made by tweezers using optical microscope and special fixators. The number of microwires was about 150. Small inclinations of wires were not critical for the considered configuration. Finally, the construction was bonded according to the scheme of P-M-P composite as presented in Figure 1, clamped mechanically and dried at 60 °C for 12 h.

The size of the rectangular PZT layer was 7 mm × 4 mm × 0.1 mm. The contacts for applying an electric field were prepared from Ag paste and were put on the sides of the PZT layers. The composite structure could be further optimized considering the dependence of the ME coefficient on the layer thickness and interface area.

The microwires were manufactured by the Taylor-Ulitovsky technique [10], in which a small amount of the metallic alloy inside a glass tube (Pyrex type in this case) was heated up to the alloy melting temperature. At this temperature glass also softened and because of rapid extraction process the molten alloy filled the glass capillary. As a result of rapid cooling, a microwire with a diameter of tens of micrometers micrometers and amorphous structure was formed. The metallic core was completely coated by glass. The effective solidification temperature of the composite microwire was about 800 °C. This temperature determines the internal thermoelastic stress when the microwire is cooled to the room temperature. The mechanical and magnetic properties of microwires were defined by the technological parameters including the cooling rate, extraction velocity, solidification temperatures, the difference in thermal expansion coefficients of glass, and metal. The latter determines the residual stress due to glass coating and the typical ratio of the expansion coefficients of glass and metal is about 0.4 [11]. The technological details along with relative properties are given in a recent review [12]. The geometrical dimensions, diameter of the core d and total diameter D of microwires, were measured by a scanning electron microscope (SEM) combined with a two-beam electron-ion super-high resolution system CrossBeam XB 540 (Zeiss, Germany).

**Figure 2:**
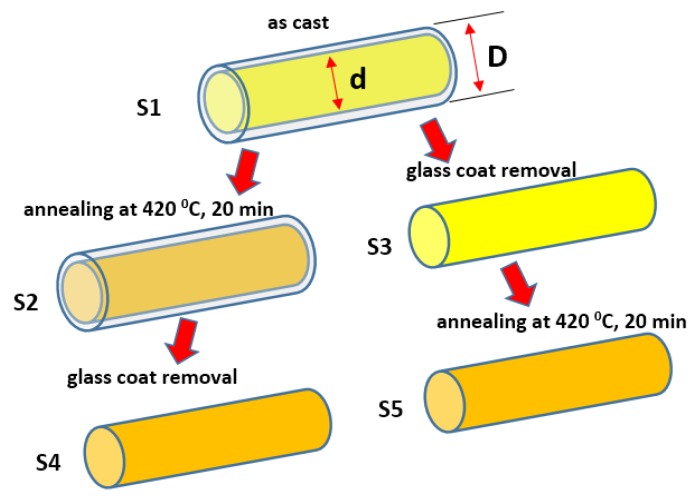
Preparation scheme for microwires.

For magnetic and magnetoelectric studies, the microwires were used as-prepared and after various post-production treatments as listed below and is illustrated in Figure 2:

(1) As-prepared in glass coat (referred to as-cast, S1);
(2) As-cast and then annealed (S2);
(3) As-cast and glass-coat removed (S3);
(4) As-cast, annealed, and then glass-coat removed (S4);
(5) As-cast, glass-coat removed, and then annealed (S5).

The glass coat was removed by a mechanical method: the wires were placed between two glass slides and a light pressure was applied. The glass coat was cracked and washed away because of insignificant adhesion to the metallic surface. Finally, the wires were cleaned in ethanol. In total, 5 types of PZT/ magnetostrictive microwire/PZT composites were prepared for the ME measurements referred to as S1-S5, respectively to the microwire type listed above and shown in Figure 2. 

The measurements of hysteresis loops of the wire samples were carried out using the vibrating sample magnetometry VSM technique at room temperature. The saturation magnetostriction coefficient $\lambda_{s}$ was measured by the small angle magnetization rotation method (SAMR) [13,14,15]. The length of the microwires for magnetostriction measurements was 10 cm. The SAMR method is known to be very sensitive for measuring negative values of magnetostriction in Co-rich amorphous wires (down to $-10^{-8}$). It was recently amended for wires with positive magnetostriction and axial anisotropy [14,15]. In this method, the wire is saturated along the axis by a dc bias field $H_{b}$ and then the magnetization is deviated from the axis by a small angle applying an AC current. This generates a voltage of the doubled frequency, the magnitude of which is kept constant when the external stress $\sigma_{ex}$ is applied by adjusting the bias field $H_{b}$. The value of $\lambda_{s}$ is defined as Equation (3):

(3) $$\lambda_{s}=-\frac{\mu_{0\;}M_{s}}{3}\frac{dH_{b}}{d\sigma_{ex}}$$

Here $\mu_{0}$ is the permeability of vacuum and $M_{s}$ is the saturation magnetization. The magnetostriction was measured by hanging a load to the wire end of 2–10 g and making a number of measurements (3–5). The average of these measurements was used for $\lambda_{s}$. The accuracy of the method is less than 5%.

**Figure 3:**
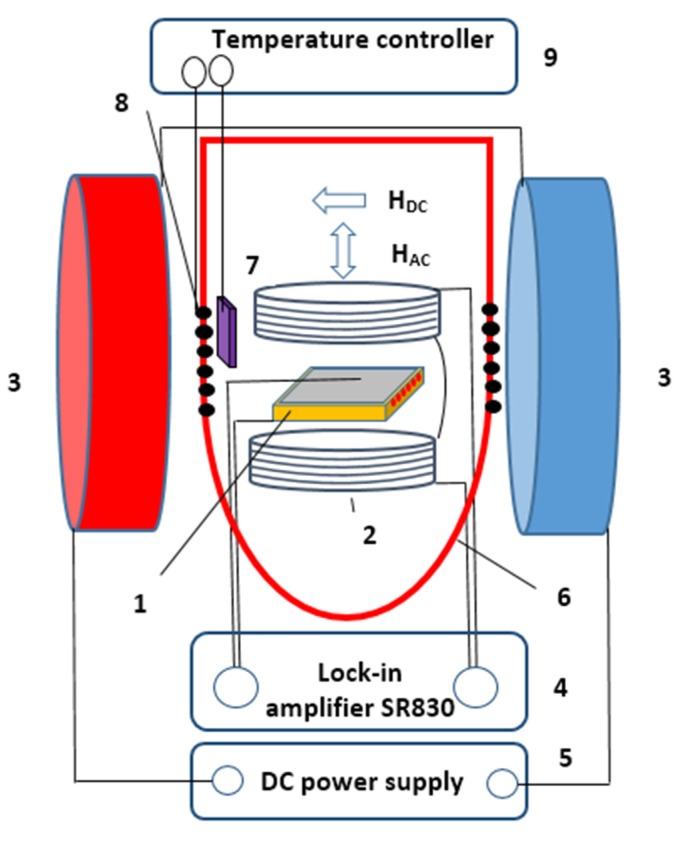
Schematics of the direct ME voltage measurement setup. (1) ME sample, (2) small Helmholtz coils for generating $H_{AC}$, (3) large Helmholtz coils for generating $H_{DC}$, (4) lock-in amplifier, (5) DC power supply, and (6)–(9) temperature control units.

The ME studies were carried out using a custom-designed setup for measuring the magnetoelectric voltage ΔV with the help of a lock-in amplifier (Stanford Research, Model SR830) in the temperature range of 170–400 K at frequencies of 0.1–100 kHz. The schematic illustration of the setup is given in Figure 3. The voltage ΔV was generated across the sample (1) subjected to an alternating magnetic field $H_{AC}$ in the presence of a DC magnetic field $H_{DC}$ [16,17]. The field $H_{AC}$ was produced by small Helmholtz coils (2) sourced by an internal generator of lock-in amplifier (4). Large Helmholtz coils (3) were used to induce the field $H_{DC}$ sourced by DC power supply (5). The amplitude of $H_{AC}$ was about 80 A/m and the DC field was varied up to 2400 A/m with a step of 79.62–159.24 A/m. The field $H_{DC}$ was applied along the wires in the plane of PZT layers and $H_{AC}$ was applied across the sandwich, that is $H_{AC}\;⊥H_{DC}$ and the voltage ΔV was measured along $H_{AC}$ (longitudinal ME effect). For ME measurements at selected temperatures, the sample was placed into adiabatic camera (6) and the system consisted of thermometer (7), heater (8) and temperature regulator (LakeShore series) (9). The ME coefficient $\alpha_{ME}$ was defined using equation (2) where ΔV is the amplitude of the induced ME voltage and ΔH is the amplitude of the AC field $H_{AC}$. The accuracy of ME coefficient measurements was less than 1%.

## 3. Results and Discussion

**Figure 4:**
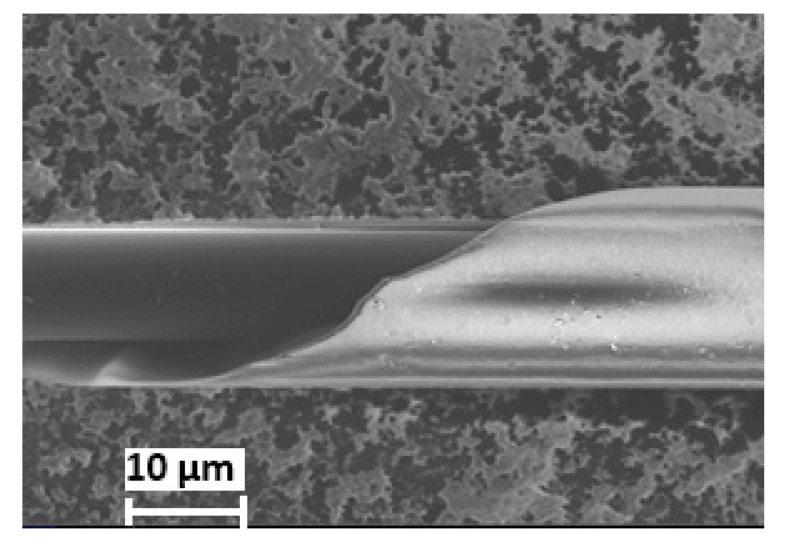
SEM image of the microwire with the composition of Fe_77.5_B_15_Si_7.5_.

Figure 4 shows a typical SEM image of the as-cast microwire in a glass coat, which was used to determine the geometrical parameters *d* = 12 μm and *D* = 26 μm. 

**Figure 5:**
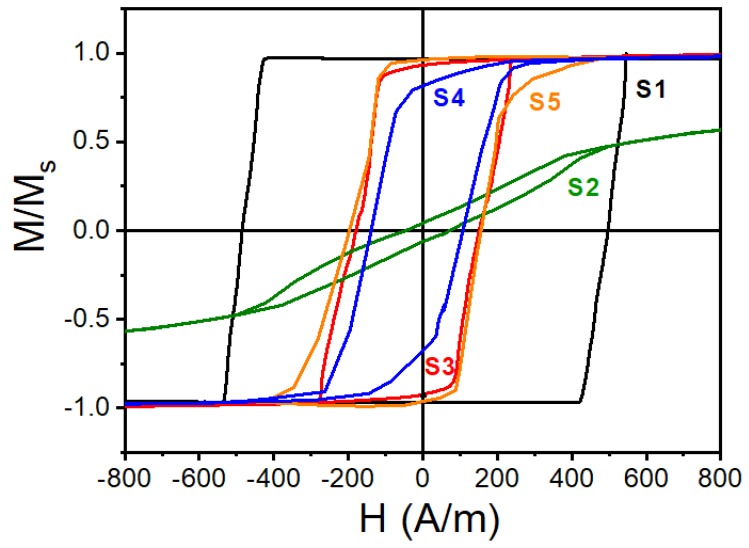
Hysteresis loops of individual microwires with composition of Fe_77.5_B_15_Si_7.5_ in as-cast state (S1) and after different treatments (S2–S5).

Figure 5 illustrates the normalized hysteresis loops for as-cast microwires (S1) and for wires after different treatments (S2–S5). S1 demonstrates a perfect rectangular loop, which is typical for Fe-rich microwires with positive magnetostriction and large internal tensile stress. Such a loop is a consequence of the magnetization reversal, which proceeds by a large Barkhausen jump [18]. The magnetic bi-stability properties are lost when the wires in glass coat are annealed (S2). This is rather unusual behavior but could be related with the transverse anisotropy induced during annealing in the presence of stress caused by glass coating [19] or due to change in the stress distribution inside the wire due to structural relaxation.

After glass removal (S3), the internal stress caused by the difference in the thermal expansion coefficients of glass and metal was released. The loop preserved a rectangular shape but the coercivity decreased from 493.6 to 167.2 A/m. There is also a portion in the hysteresis loop that is associated with the rotational processes in the outer shell caused by micromagnetic structure changes [20]. The comparison between the magnetic properties of glass-coated (S1) and glass-removed (S3) microwires is presented in Table 1. The saturation magnetostriction $\lambda_{s}$ increased by an order of magnitude after glass removal. This could be explained by stress-dependence of $\lambda_{s}$ [21] and substantial decrease in the internal stress when the thermoelastic stress caused by glass coating was released.

When the glass was removed after the as-cast wire was annealed (S4) the bi-stability was restored but the rotational portion of the magnetization loop was still present. There was some change in the hysteresis loops of wires (S4) and (S5), which was annealed after glass removal. In the former case, the wire annealing was in the presence of the glass shell exerting a stress during annealing so some transverse anisotropy remained. We will further demonstrate that the ME effect increases when the annealed wires without glass are used (although quite similar for wires annealed either before glass removal or after). The wire magnetic behavior could be further refined by changing the post-production treatments [20,21].

**Figure 6:**
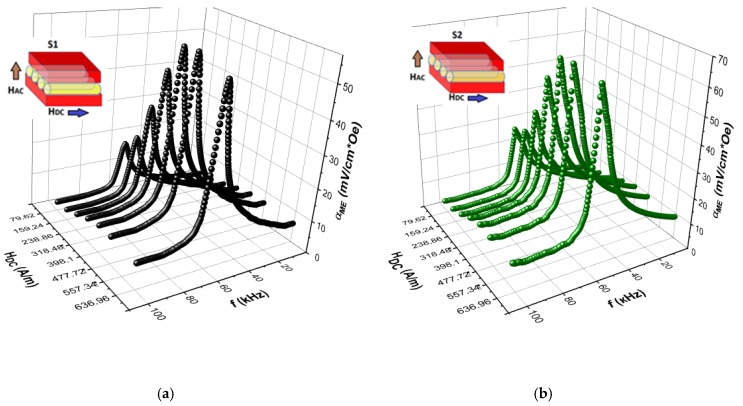
Frequency dependencies of the ME voltage coefficient for different DC bias field $H_{DC}$ for sandwiches with glass coated microwires (as-prepared—S1 (a) and after annealing—S2 (b)).

**Figure 7:**
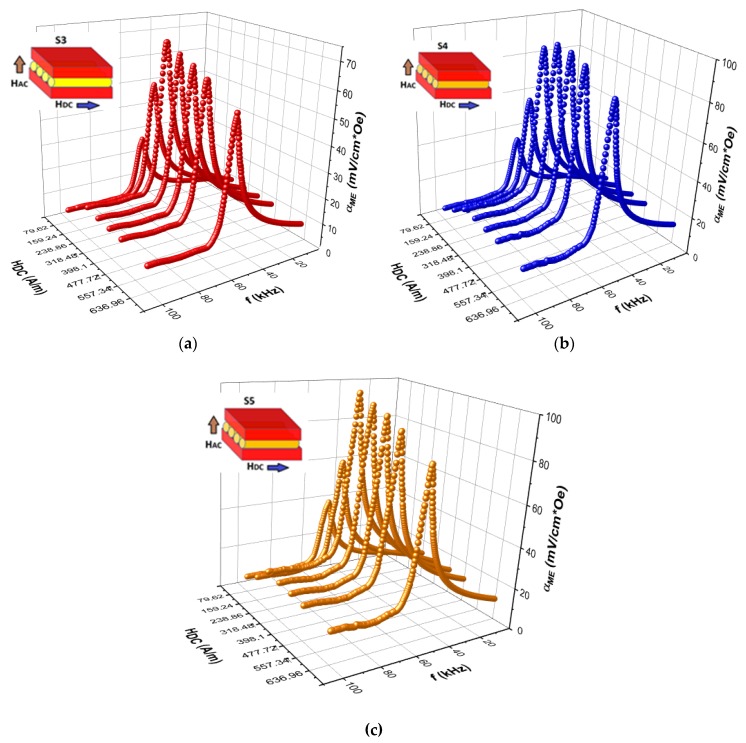
Frequency dependencies of the ME voltage coefficient for different DC bias field $H_{DC}$ for sandwiches with microwires after glass removal (S3 (**a**), S4 (**b**), S5 (**c**); S4, and S5 were also annealed before and after glass removal, respectively).

The dependencies of the ME coefficient $\alpha_{ME}$ on the frequency of modulating magnetic field $H_{AC}$ for different DC magnetic fields $H_{DC}$ measured for all sandwich samples involving the microwires with different treatments (S1–S5) at room temperature are shown in Figure 6 and Figure 7.

The peaks in ME voltage are associated with the length mechanical resonance of the magnetic wires typical for composite structures [22,23,24] and obviously are attributed to the fundamental vibration of the sample caused by the magnetostrictive response in the magnetic layer under the AC magnetic field. As seen from Figure 6 and Figure 7, the resonant frequencies were not significantly changed with increasing $H_{DC}$. The ME voltage demonstrated small values out-off resonance, the resonance ME voltage shows a peak at the DC magnetic field close to the wire magnetization saturation. This behavior corresponds to the field dependence of the piezomagnetic parameter q=∂λ/∂H [25]. The weak ME effect in the composite with glass-coated microwires was caused by poor connectivity between the phases through glass coat. Moreover, the glass coat played a role of a passive layer, which led to the damping of vibrations induced by the magnetic layer. Ultimately, the above factors led to a decrease in the ME effect.

Removing the glass coat slightly increased the maximum of the ME coefficient and the resonance frequency decreased. According to the effective medium approximation, the resonance frequency was proportional to the square root of the ratio of the average Young modulus Y and density ρ: $f_{res}\sim \sqrt{Y/\rho }$ [26,27]. When the glass was removed, the frequency was decreased by about 10%, which could be related with an increase in density. The ME coefficients considerably increased (more than 60%) for annealed wires (before or after glass removal) but the resonance frequency remained almost the same. 

**Figure 8:**
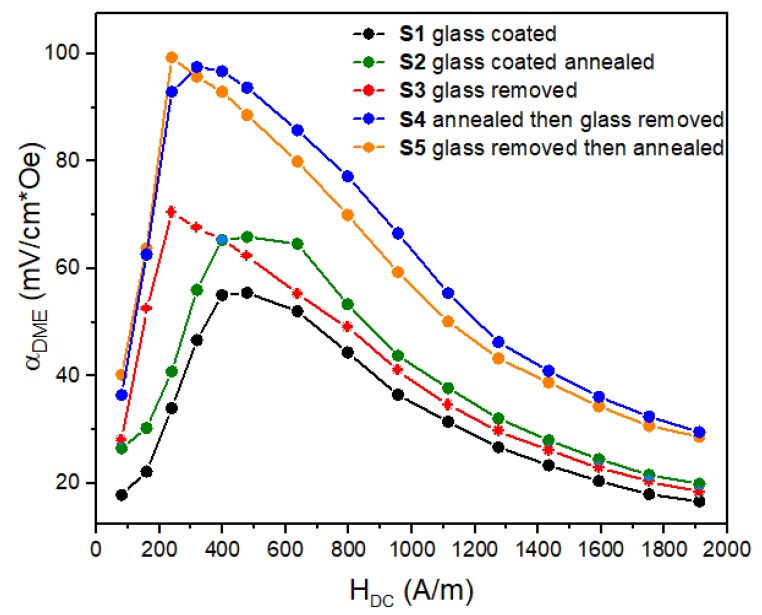
Dependencies of the ME voltage coefficient $\alpha_{ME}$ on DC magnetic field $H_{DC}$ field for S1-S5 composites at resonance condition.

In Figure 8, the comparison of the magnetic field dependences of the ME coefficient $\alpha_{ME}$ for S1-S5 composites is presented at the resonant frequency. The ME voltage coefficients for all the samples show its maximum in the field range of 240–400 A/m and depended on the microwire preparation protocol. For higher field, when the microwires were saturated along the axis the perpendicularly applied field $H_{AC}$ could not produce a significant vibration, therefore, the ME coefficient decreased when $H_{Dc}$ exceeded the saturation field.

The maximum of the ME effect was observed utilizing the annealed wires without glass. Slightly better behavior of $\alpha_{ME}$ was observed for composites with wires annealed after glass removal, which completely avoided the presence of the induced anisotropy and residual stress. 

The ME parameters deduced from measurement data depicted in Figure 6, Figure 7 and Figure 8 are collected in Table 2. It is seen that at the resonance conditions the ME coefficient of wire-composites increased when using the wires without glass (S3–S5). Firstly, the magnetostriction coefficient increased due to internal stress relief when glass was removed (see Table 1). Secondly, there was attenuation of the vibrations in the glass coat. The annealing treatment (before or after glass removal), which released the residual stress from quenching, increased the magnetization rotation contribution into the magnetization reversal process and further enhanced the ME effect: the ME coefficient increased almost twice becoming nearly 100 mV/cm × Oe. This value is still small comparing the previous results obtained for best layered composites [1,2,3,4,28]. For example, the maximum of the ME effect in 4 μm thick PZT film deposited on amorphous magnetostrictive Metglass foil (FeBSi) was about 3 V/cm × Oe in a DC magnetic field 4800 A/m [29]. This illustrates the role of not only the piezomagnetic and piezoelectric characteristics of the composite’s components, but also the impact of the connectivity type of the connection between the layers. The deposition excluded the effects related with mechanical losses on adhesive boundaries and led to the enhancement of the ME effect. Some other factors including the volumetric ratio of magnetic and piezoelectric layers, perfection of interfaces between the layers and level of bonding also strongly affect the ME voltage [28,29,30,31]. However, in the present case the thickness of the magnetostrictive layer was small compared to that of the piezoelectric layer. The interfaces between the layers of different phases were also not perfectly bonded together. The imperfect interfaces definitely affected the mechanical and magnetoelectric properties of the developed ME composites. In our case, a simple fabrication process as demonstrated in Figure 1 was not capable of producing good phase interfaces. Nevertheless, we demonstrated a high potential of amorphous magnetostrictive wires for applications in ME composites. 

## 4. Conclusions

A new ME sandwich structure comprising glass-coated amorphous Fe_77.5_B_15_Si_7.5_ microwires as a magnetostrictive layer were fabricated and studied. The enhancement of the ME voltage due to glass coat removal and annealing (either before glass-coat removal or after) was demonstrated. This was justified by the increase in the magnetostriction parameter and its field gradient along with the improvements in interface bonding. After optimal annealing of microwires (at 400 °C for 20 min) and glass removal the ME coefficient increased up to 100 mV/cm × Oe. This maximum was seen in weak magnetic fields about 240 A/m at electromechanical resonance conditions. The proposed ME composites had the potential for low-field applications. In particular, due to the sensitivity of soft magnetic materials to low magnetic field, this approach can be used for the design of new self-biased magnetoelectric composites that provide large ME coupling under an external AC magnetic field in the absence of a DC magnetic field [32,33,34]. Further improvements in the ME composites with amorphous magnetostrictive wires are related with the technology development in terms of increasing the volumetric fraction of the wires and improvements in interface bonding [35]. 

## Figures and Tables

**Table 1:** Geometrical and magnetic parameters of glass-covered (S1) and glass removed (S3) Fe_77.5_B_15_Si_7.5_ microwires. Designations: $H_{c}$ is the coercive field, $M_{r}$ is the remnant magnetization, $M_{s}$ is the saturation magnetization, and $\lambda_{s}$ is the saturation magnetostriction.

Composition	*d*, μm	*D*, μm	ρ=d/D	$H_{c}$, A/m	$M_{r}/M_{s}$	$\lambda_{s}$	Condition
Fe_77.5_B_15_Si_7.5_	12	26	0.46	493.6	0.99	$\left(1.9\pm 0.1\right)\times 10^{-6}$	(S1) As-cast, with glass
				167.2	0.94	$\left(5.4\pm 0.3\right)\times 10^{-5}$	(S3) Without glass

**Table 2:** Magnetoelectric parameters of composites with glass-covered and glass removed Fe_77.5_B_15_Si_7.5_ microwires (* in parentheses, the value of the DC magnetic field at which $\alpha_{ME}$ has a maximum is given).

Microwire	Glass Coated		Glass Coat Removed		
	As-Cast *S1*	Annealed *S2*	Glass Removed *S3*	Annealed then Glass Removed *S4*	Glass Removed then Annealed *S5*
f_res_, kHz	57.9	55.2	52.5	51.9	51
α_ME_ (max), * mV/cm×Oe	55.49 (477.7 A/m)	65.9 (477.7 A/m)	70.5 (238.8 A/m)	97.55 (318.48 A/m)	99.32 (238.8 A/m)
